# A case report of a ruptured Meckel's diverticulum with ectopic gastric and pancreatic tissue with negative computed tomography

**DOI:** 10.1016/j.ijscr.2021.105994

**Published:** 2021-05-21

**Authors:** Marcos Rosado, Thomas Serena, John Pui, John Parmely

**Affiliations:** a Department of General Surgery, Beaumont, Health Farmington Hills, MI, USA; b Department of Pathology, Beaumont, Health Farmington Hills, MI, USA

**Keywords:** Meckel diverticulum, Ectopic gastric and pancreatic tissue, Case report

## Abstract

**Introduction:**

A Meckel's diverticulum is a rare but known cause of an acute abdomen and can often be confused for acute appendicitis on physical examination. It is caused by an incomplete closure of the omphalomesenteric duct. It is present in 2% of the population and only 2% of those patients are symptomatic.

**Case presentation:**

This is the case of a sixty-four-year-old male presented to the surgical clinic at request of his primary care physician with concern for acute appendicitis. The patient had a CT A/P with IV contrast performed two days prior to his office visit for the same pain which was non-diagnostic. The patient was taken to the operating room and found to have Meckel's Diverticulitis which was managed by laparoscopic hand-assisted small bowel resection and anastomosis. The patient had an uncomplicated postoperative course. Pathology demonstrated ulcerated gastric mucosa and pancreatic tissue.

**Discussion:**

Symptomatic Meckel's diverticulum is managed with small bowel resection versus diverticulectomy based on characteristics of the diverticulum. The most common type of ectopic tissue is gastric followed by pancreatic. It is rare to find both types of tissue together.

**Conclusion:**

This case describes an unusual case of a rare acute surgical pathology with non-diagnostic imaging and labs. This case also describes an exceedingly rare histopathology of a Meckel's Diverticulum with the presence of both ectopic gastric and pancreatic tissues.

## Highlights

• Meckel's Diverticulum in adults commonly presents with inflammation, small bowel obstruction and perforation.
• The recommendation for treatment of a symptomatic Meckel's diverticulum is surgical resection.
• Meckel's Diverticulum contain ectopic tissue, most commonly gastric and pancreatic tissue but rarely together.

## 1 Introduction

A Meckel's diverticulum is the most common congenital abnormality of the small intestine and is caused by the incomplete closure of the omphalomesenteric duct. It has been estimated to be present in two to 4% of the population [1]. It was first described by German surgeon Guilhelmus Fabricius Hildanus in 1598. The name comes from Johann Friedrich Meckel who described this pathology and proposed its embryological origins in 1809 [2]. While rarely symptomatic, it should always be part of the differential diagnosis of a patient presenting with right lower quadrant abdominal pain. When symptomatic, a Meckel's Diverticulum should be treated with surgical resection by performing a diverticulectomy or small bowel resection. This report was written in accordance with the SCARE 2020 criteria [3].

## 2 Case presentation

This case report presents a sixty-four-year-old Caucasian male with a three-month history of intermittent right upper quadrant and epigastric abdominal pain. The patient previously underwent an extensive negative work-up for biliary pathology in addition to multiple emergency room visits without any diagnoses. The patient went to his primary care provider two days prior to surgical evaluation where he was noted to have a temperature of 100.4 °F (38.0 °C) and computed tomography (CT) of the abdomen and pelvis with IV contrast was ordered due to concern for appendicitis. There was no acute pathology identified on this scan. The patient has a past medical history of coronary artery disease, hypertension, type 2 diabetes mellitus, obstructive sleep apnea, and GERD. Past surgical history was significant for a prior umbilical hernia repair with mesh. The patient had no known allergies, no significant family history and no significant social history. Due to concern for acute appendicitis the patient was referred to the surgical clinic for further evaluation.

At the time of surgical evaluation, the patient reported a new, three-day onset of localized right lower quadrant abdominal pain. Review of systems revealed associated nausea and anorexia without vomiting, change in bowel habits, or rectal bleeding. On physical examination, the patient's vital signs were within normal limits. The patient appeared mildly uncomfortable but in no acute distress. His abdomen was obese with tenderness to palpation in the right lower quadrant as well as localized right lower quadrant percussion tenderness, rebound tenderness and a positive McBurney's point. Laboratory work-up was performed revealing a normal white blood cell count of 5.6 bil/L, electrolytes within normal limits, and a mildly elevated C-reactive protein level at 45 mg/L. The patient's CT scan had been performed at an outside facility and was unable to be personally reviewed but the report was available which reported no acute or abnormal intra-abdominal pathology.

Based on the patient's physical exam and elevated CRP level decision was made to take the patient to the operating room for diagnostic laparoscopy with clinical concern for appendicitis. Other diagnoses in our different diagnosis were Meckel's Diverticulitis, inflammatory bowel disease, diverticulitis, perforated viscus, or an intra-abdominal malignancy. The patient received antibiotics and subcutaneous heparin preoperatively. The abdomen was sterilized using Chloroprep solution and was pneumoperitoneum was created using the Veress needle technique. Upon entry the omentum was noted to be in the right lower quadrant and inflamed in appearance. The appendix was identified and found to be normal in appearance. The rest of the abdomen was visually inspected with no immediate obvious pathology. Knowing the appendix was normally the decision was made to inspect the small intestine and remainder of abdomen to rule out other acute pathology. There was no evidence of inguinal or abdominal wall hernia. On inspecting the small intestine an inflammatory mass was identified with omentum tightly adherent to it located approximately 2 ft from the ileocecal valve. This mass was in the right lower quadrant of the abdomen. The omentum was carefully dissected free and the intestine closely inspected with an inflamed Meckel's diverticulum identified. The remaining small intestine was run with no further pathology identified. Based on a wide base and short length of the diverticulum the decision was made to perform a small bowel resection with primary anastomosis. The midline port was extended and a gel port was inserted. The Meckel's diverticulum was pulled through the gel port and an external bowel resection with primary stapled side to side anastomosis was performed.

**Image 1:**
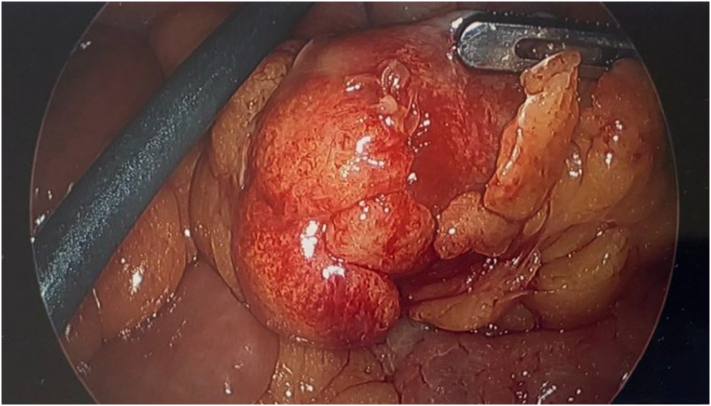
Intraoperative photograph demonstrating an inflammatory mass discovered while running the small intestine.

**Image 2:**
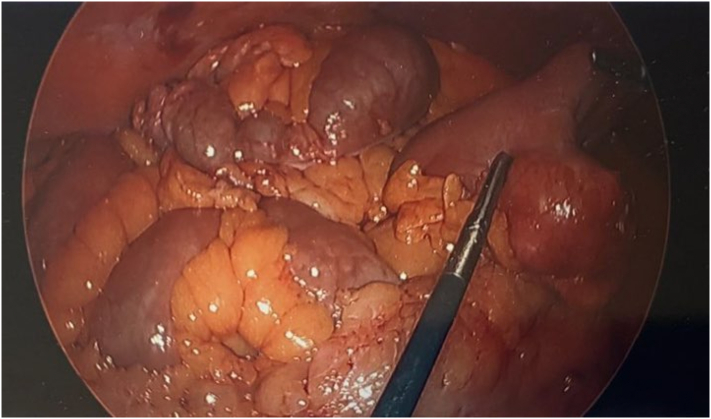
Intraoperative photograph demosntrating a Meckel's Diverticulum that is acutely inflamed.

Intraoperative Imaging: Image 1, Image 2 revealing mobilization of a large mass protruding from the small intestine later identified as a Meckel's Diverticulum.

The patient recovered well from surgery and was discharged three days postoperatively. Surgical pathology demonstrated benign small bowel with a Meckel's diverticulum with features of acute inflammation and rupture. Further pathological analysis demonstrated an ulcerated benign gastric-type mucosa as well as benign pancreatic tissue within the diverticulum, without evidence of malignancy. In office follow-up the patient was found to have an incisional wound infection of his midline incision which was treated with antibiotics and local wound care. A chronic draining wound developed and five months after his original surgery the wound was re-excised. He healed well afterwards with no further complication.

**Image 3:**
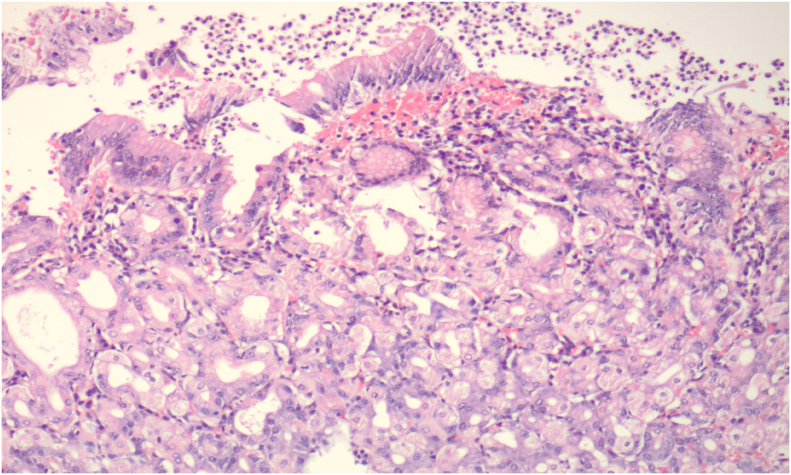
Distorted gastric body type mucosa adjacent to ulcerated diverticulum mucosa. Note the eroded surface foveolar epithelium with associated acute inflammation, as well as the underlying gastric glands with parietal and chief cells. Hematoxylin and eosin, original magnification 100x.

**Image 4:**
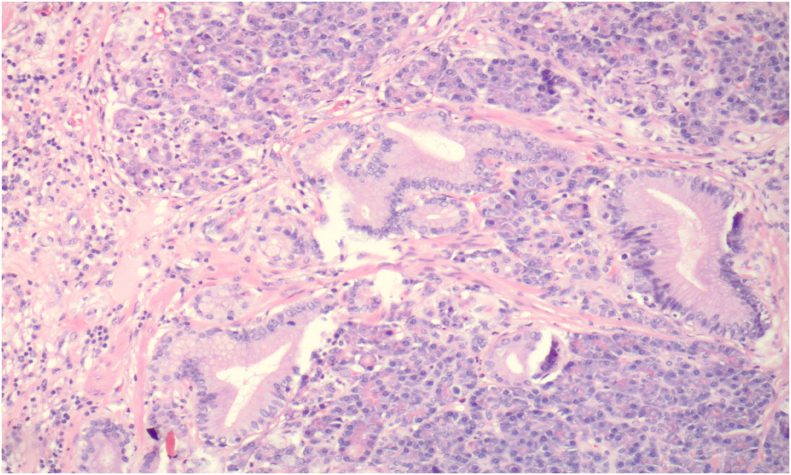
Pancreatic exocrine type mucosa in the wall of the diverticulum. Lobules of pancreatic acinar cells surround mucinous ductal structures recapitulating extrabiliary bile ducts. Hematoxylin and eosin, original magnification 100x.

Histological Evaluation: Image 3. Distorted gastric body type mucosa adjacent to ulcerated diverticulum mucosa. Note the eroded surface foveolar epithelium with associated acute inflammation, as well as the underlying gastric glands with parietal and chief cells. Hematoxylin and eosin, original magnification 100×. Image 4. Pancreatic exocrine type mucosa in the wall of the diverticulum. Lobules of pancreatic acinar cells surround mucinous ductal structures recapitulating extrabiliary bile ducts. Hematoxylin and eosin, original magnification 100×.

## 3 Discussion

The presentation of a Meckel's diverticulum varies between the pediatric and adult populations. Pediatric populations most commonly present with gastrointestinal bleeding and obstruction [3]. In fact, the most common etiology of bowel obstructions in pediatric populations are intussusception or volvulus secondary to Meckel's diverticulum [4]. In adults, patients most commonly present with obstruction, inflammation, diverticulitis, and perforation. The multitude of symptoms can vary further if there is presence of ectopic tissue, the most common types being gastric or pancreatic tissue. A clear association has been demonstrated between ectopic gastric tissue in Meckel's diverticulum and gastrointestinal bleeding. In a meta-analysis performed by Carlioz reviewing 8389 cases of Meckel's that were operated on for gastrointestinal bleeding, 98% of patients were found to have ectopic gastric tissue [5]. Other rare presentations of Meckel's diverticulum are a Littre's hernia, a hernia sac containing a Meckek's diverticulum. While classically described as occurring in an inguinal hernia, it can occur in a femoral hernia, umbilical hernia, and has even been described in a transthoracic hernia [6,7]. Another incredibly rare presentation is an enterovesical fistula, with few cases reported in the literature [8]. Lastly, 0.5–1.9% of patient's may experience oncological progression with mutation into a tumor [10].

Once a patient is diagnosed with symptomatic Meckel's diverticula, the standard of care is definitive surgical intervention. Surgical options are diverticulectomy, wedge resection, or segmental bowel resection. These procedures can be performed either open or laparoscopically depending on the surgeons comfort level. In pediatric surgery literature, the outcomes of laparoscopic vs open surgery have been found to be equivalent in regards to length of stay, postoperative complications, reoperations, and readmissions [9]. Konstantinos et al. published a recent review on surgical approaches for Meckel's diverticulum based on the type of pathology and size of the diverticulum. In patients with gastrointestinal bleeding, the majority of cases will have ectopic gastric tissue. As described by Varcoe et al., patient's with a long diverticula (height-to-diameter ratio >2) have ectopic tissue present at the body and tip of the diverticulum while patients with a short diverticula have a wide distribution of ectopic tissue including the base of the diverticulum. They also found that the external appearance of the diverticulum did not predict the presence of ectopic gastric mucosa and suggested surgical decision making not be made solely on the external appearance of the diverticulum. [11] Wedge or segmental bowel resection should be performed in order to ensure all ectopic gastric tissue is resected. If the patient presents with simple diverticulitis, a diverticulectomy can be performed if a long diverticulum is present or a wedge or segmental resection if a short diverticulum is present. If the pathology is complicated (intestinal obstruction, diverticulitis with perforation, tumor) then wedge or segmental resection should be performed [12].

Based on the “Rule of 2's” and in review of literature, approximately 2% of the population has Meckel's diverticulum and 2% of those patients will become symptomatic. Based on this, the majority of Meckel's diverticulum that are present are asymptomatic and surgeons will often encounter them while operating on patients for a different reason. The decision to resect an incidentally discovered diverticula has been debated with several studies reviewing the outcomes of incidentally resected diverticula. In a review by Zani et al., it was found that there was a statistically significant higher postoperative complication rate following resection of an incidentally identified Meckel's [13]. In contrast to this, a retrospective study by Zulfikaroglou found no significant difference between symptomatic and asymptomatic patients with regards to postoperative complications [14]. In an article by Konstantinos et al., they identified four risk factors from prior reviews that were considered high risk factors for developing future complications from MD. These risk factors are 1) patient age <50 years, 2) male sex, 3) diverticulum length > 2 cm, and 4) ectopic or abnormal features within a diverticulum wall. For these incidentally identified diverticulum, the authors recommend diverticulectomy for long diverticula and wedge or segmental resection for short diverticula.

The omphalomesenteric duct is made up of pluripotent tissue that can mature into several different types of tissue in a MD. While the tissue is typically ileal tissue, ectopic tissues can develop within the diverticulum. The most common tissue is gastric tissue which is present in up to twelve to 26% of cases. The second most common type of ectopic tissue is pancreatic tissue, occurring in roughly two to 3% of patients. Other less common types of ectopic tissue are duodenal, colonic, endometrial, and hepatobiliary [3]. The final pathology in this case report identified two types of ectopic tissue with the presence of both gastric and pancreatic tissues. This finding is exceedingly rare with two reviews describing this dual pathology with an incidence of 0.8–2.6% [15,16]. The gastric mucosa was noted to be ulcerated and was likely the cause of the patient's diverticulitis.

This case report is also unusual in the patient's presentation. The patient had a several month history of right sided abdominal pain, initially worse in the right upper quadrant and epigastrium. The patient was evaluated for biliary pathology and reported a negative workup. Two days prior to presentation to the surgical clinic, the patient had developed worsening right lower quadrant abdominal pain and had a CT of the abdomen and pelvis that was performed with IV contrast that was negative for findings of appendicitis, diverticulitis, or intra-abdominal inflammation. Modern computed tomography imaging is able to detect a broad amount of intra-abdominal pathology so a patient with negative imaging and continued severe abdominal pain can present the physician with a diagnostic dilemma. In the setting of Meckel's diverticulitis, imaging findings will demonstrate a blind-ended tubular structure with wall thickening and enhancement with surrounding fat stranding. These findings can be seen in the case of acute appendicitis so the diagnosis of Meckel's diverticulitis on imaging requires the finding of a normal appendix [17]. It is unclear why this patient's CT imaging did not demonstrate any acute intra-abdominal findings. Given that the patient's histopathology demonstrated findings of ruptured Meckel's Diverticulitis it is possible the CT scan was performed prior to rupture and development of significant findings able to be seen on the CT scan.

## 4 Conclusion

Meckel's Diverticulum is an uncommon presentation of right lower quadrant abdominal pain but should always be part of a physician's differential diagnosis. Physicians tend to rely more on radiographic imaging and laboratory findings and pay less attention to the physical examination to help elucidate a patient's abdominal pathology. This case is unique as the preoperative imaging was normal and raised no suspicion of the underlying acute surgical pathology. The main concerning feature in this patient was the physical examination prompting a diagnostic laparoscopy searching for appendicitis and instead finding acute Meckel's Diverticulitis. As stated by Dr. Charles Mayo in 1933, “Meckel's diverticulum is often suspected, always looked for and rarely found” [19]. This case is also unique with regards to the pathologic analysis of the Meckel's diverticulum which demonstrated both gastric and pancreatic ectopic tissues, an exceedingly rare finding. This case demonstrates the importance of a good physical examination and reinforces the concept of developing a differential diagnosis in order to accurately diagnose and treat acute abdominal pain.

## Provenance and peer review

Not commissioned, externally peer-reviewed.

## Sources of funding

Beaumont Farmington Hills Department of Medical Education.

## Ethical approval

Not required for case report with no patient identifiers as there is minimal risk to the patient.

## Consent

Written informed consent was obtained from the patient for publication of this case report and accompanying images. A copy of the written consent is available for review by the Editor-in-Chief of this journal on request.

## Research registration

Not applicable.

## Guarantor

Marcos Rosado.

## CRediT authorship contribution statement

Marcos Rosado: Primary author of case report, performed literature review.

Thomas Serena: Contributing author and editor of case report.

John Pui: Provided pathology slides and commentary, reviewed paper.

John Parmely: Primary surgical attending, reviewed paper.

## Declaration of competing interest

None.
